# Study on mechanism of action of total flavonoids from Cortex Juglandis Mandshuricae against alcoholic liver disease based on “gut-liver axis”

**DOI:** 10.3389/fphar.2022.1074286

**Published:** 2023-01-11

**Authors:** Huiru Liu, Wenwen Meng, Dongsheng Zhao, Zhihui Ma, Wenguang Zhang, Zhi Chen, Zhengguo Li, Pan Zhao

**Affiliations:** ^1^ College of Pharmacy, Shandong University of Traditional Chinese Medicine, Jinan, Shandong, China; ^2^ Jining Food and Drug Inspection and Testing Research Institute, Jining, Shandong, China

**Keywords:** alcoholic liver injury, cortex juglandis mandshuricae, total flavonoids, hepatoprotective effect, gut-liver axis

## Abstract

The objective of this study was to investigate the effects and molecular mechanisms of total flavonoids from Cortex Juglandis Mandshuricae (TFC) on preventing alcohol-induced chronic liver injury and regulating gut microbiota in mice. The results showed that oral administration of TFC significantly attenuated alcoholic liver injury in mice. TFC improved lipid accumulation in mice with chronic alcoholic liver injury through activation of the AMPK/PPARα pathway. In addition, TFC maintained the integrity of the intestinal barrier in alcoholic mice, reducing endotoxin leakage from the intestine and further inhibiting the TLR4/NF-κB inflammatory pathway. More importantly, TFC regulated the intestinal microbiota composition and certain bacteria, including *Akkermansia muciniphila, Lactobacillus* and others. At the same time, reduced levels of short-chain fatty acids due to alcohol consumption were restored. In summary, TFC upregulated AMPK/PPARα signaling pathway to improve hepatic fat accumulation and oxidative stress; TFC positively regulated intestinal flora composition to reduce intestinal disorders caused by alcohol consumption, and further inhibited alcohol-induced inflammatory responses through the intestinal-liver axis. The above findings may be the mechanism of TFC’s pharmacological effects against alcoholic liver injury.

## 1 Introduction

Excessive alcohol consumption is one of the leading causes of alcoholic liver disease (ALD), a disease that affects human health worldwide. Globally, an estimated 741,300 of all new cancer cases in 2020 have been reported to be due to alcohol consumption (Rumgay et al., 2021). Alcoholic liver disease refers to a wide range of disorders, including early liver steatosis and steatohepatitis, advanced liver fibrosis and cirrhosis, which may eventually develop into hepatocellular carcinoma in severe cases (Williams et al., 2014). Despite the growing body of research into the mechanisms of ALD, clinical strategies for the prevention and treatment of ALD remain inadequate; it is urgent to find effective and safe drugs to prevent and treat ALD.

Recent research into gut microbiology has provided a wealth of experimental and clinical data that suggests that the gut microbiota plays an important role in the pathogenesis of ALD. The gut microbiome is a diverse ecosystem of bacteria, fungi and viruses and other microbes. Alcohol consumption directly contributes to microbial dysbiosis in the gut, altering the diversity and composition of the intestinal biota and causing disturbances in the intestinal environment. Alcohol also modulates the composition of the gut microbiota by decreasing concentrations of most short-chain fatty acids (SCFAs); other than acetic acid, a metabolite of ethanol, the levels of all other SCFAs are lowered in the gut following alcohol intake (Xie et al., 2013). Alcohol also structurally modifies the gut tissue, which impacts the functioning of connections between the gut and the liver. In fact, the gut-liver axis provides the main pathway for the development and progression of ALD, as alcohol intake disrupts the integrity of the intestinal barrier and reduces levels of the associated tight junction proteins ZO-1, occludin and claudin-1 (Cho and Song, 2018). The dysregulated intestinal flora can produce large amounts of the endotoxin lipopolysaccharide (LPS) (Keshavarzian et al., 2009); because of the associated increased permeability, the excess LPS and other bacterial products can be transferred to the bloodstream *via* the portal vein and proceed to the liver. In the liver, LPS activates Kupffer cells and macrophages by binding to toll-like receptor 4, which activates the nuclear factor-κB (NF-κB) pathway. This pathway induces the production of inflammatory chemokines, including tumor necrosis factor (TNF)-α, interleukin (IL)-1β and IL-6 (Szabo, 2015).

Abnormalities in lipid metabolism have long been recognized as an important factor in the pathogenesis of ALD. Peroxisome proliferator-activated receptor alpha (PPARα) is a nuclear hormone receptor that plays an important role in lipid metabolism by regulating the expression of many genes involved in the transport and oxidation of free fatty acids. Alcohol inhibits fatty acid oxidation in part by suppressing the transcriptional activity and DNA binding capacity of PPARα, and the alcohol metabolite acetaldehyde can downregulate PPARα directly or indirectly through other pathways (Li et al., 2014). In addition to its effects on PPARα, alcohol also inhibits fatty acid synthesis by inhibiting the phosphorylation of 5′-AMP-activated protein kinase (AMPK) and, leading to accelerated progression of hepatic steatosis (You et al., 2004). Therefore, the regulation of intestinal flora disorders and the inhibition of abnormal lipid metabolism would be predicted to improve the liver damage caused by alcohol intake.

From a therapeutic point of view, Traditional Chinese Medicines (TCM) are of growing interest as promising approaches that are recognized to show better efficacy and fewer side effects than synthetic drugs (Li, 2020). In particular, there is compelling evidence supporting the beneficial effects of Chinese herbal extracts on the prevention and treatment of ALD (Lu et al., 2012). Cortex Juglandis Mandshuricae (CJM), the bark of Juglans mandshurica Maxim was first published in Kaibao Materia as a popular Chinese folk medicinal plant used to fight cancer and treat diarrhea and dysentery (Hou et al., 2011). CJM enters the liver meridian and clears heat and dampness, drains the liver and brightens eyesight, effects that are consistent with an impact on ALD. Phytochemical studies have shown that the main pharmacological components in CJM are flavonoids, quinones, and phenols (Si et al., 2008). Flavonoids are a class of natural product that have a wide range of pharmacological activities, such as regulating glucose and lipid metabolism, enhancing liver fat decomposition and preventing liver toxicity, lowering inflammation, inhibiting hypertension, and protecting the cardiovascular system.

Modern pharmacological studies show that total flavonoids from Cortex Juglandis Mandshuricae (TFC) has multiple effects, including liver protection, anti-oxidation, and anti-tumor, and it is used clinically the treatment of acute and chronic hepatitis and liver cancer and other diseases (Sun et al., 2014). Previous studies have reported the protective effect of TFC on non-alcoholic liver injury (Zhao et al., 2015). However, the protective effect of TFC on ALD and the potential role of the modulation of gut microbiota by TFC in the prevention and management of ALD needs to be further studied.

In this study, we explored the mechanism by which TFC prevents alcoholic liver injury through the “gut-liver” axis. We first established a mouse model of alcoholic liver injury to reveal the protective mechanism of TFC against ALD. Furthermore, we used 16s rRNA analyses to detect gut microbiota, measure the expression of intestinal barrier-related genes and explore the possible effect of TFC on the enteric-liver axis through the LPS-TLR4-NF-κB inflammatory response pathway. This research is intended to elucidate the underlying mechanism by which TFC exerts its hepatoprotective effect and inhibits lipid metabolism abnormalities based on the “gut-liver” axis model.

## 2 Materials and methods

### 2.1 Chemicals and reagents

Cortex Juglandis Mandshuricae (bark) were purchased from Anguo City Changda Chinese Herb Beverage Co. And authorized by professor Hongyan Liu of Shandong University of Traditional Chinese Medicine. The commercial kits, including aspartate aminotransferase (AST), alanine aminotransferase (ALT), total triglyceride (TG), total cholesterol (TC), superoxide dismutase (SOD), glutathione (GSH), catalase (CAT), and malondialdehyde (MDA) were purchased from Nanjing Jiancheng Bioengineering Institute (Nanjing, Jiangsu, China). The commercial kits, including lipopolysaccharide (LPS), tumor necrosis factor-α (TNF-α), interleukin-6 (IL-6), interleukin-1β (IL-1β) were purchased from Jiangsu Jingmei Biological Technology. RIPA lysate, PMSF (100 mM), phosphorylated protease inhibitor, protein quantification (BCA) assay kit, nuclear and plasma protein extraction kit, polyvinylidene fluoride (PVDF) membrane (0.45 μm), primary antibody, secondary antibody, skimmed milk powder and enhanced chemiluminescence reagents (ECL) were purchased from Wuhan Xavier Biotechnology Co. Acetic acid, propionic acid, butyric acid, isobutyric acid, valeric acid and isovaleric acid were purchased from Chengdu Kromax Biotechnology Co.

### 2.2 Extraction and purification of TFC extract

The crushed of CJM (bark) were extracted with 70% aqueous ethanol (solvent and sample ratio was 15:1, v/w) under heat and reflux for two times (1.5 h for each). Then, the filtered solutions were gathered to obtain the alcoholic extract of CJM. Then, the filtrates were combined and recovered under reduced pressure to obtain the crude extract of CJM. The pH of the crude extract was adjusted to four and the sample was applied to HPD-750 macroporous resin. Then, 70% ethanol was used for elution, and the collected eluate was concentrated under reduced pressure and freeze-dried to obtain the total flavonoid powder of CJM, which was set aside.

### 2.3 Animals and experimental design

Eight-week-old male C57BL/6 mice weighing 20–22 g, were obtained from Beijing Vital River Laboratory Animal Technology Co., Ltd. (Beijing, China). All mice were housed under standard conditions (12 h light: dark cycle, temperature 22°C ± 2°C, humidity 55 ± 10%). All animal procedures were performed in accordance with the Guidelines for the Care and Use of Laboratory Animals of Shandong University of Traditional Chinese Medicine and approved by the Animal Ethics Committee at Shandong University of Traditional Chinese Medicine.

After 1 week of acclimatization, the mice were divided into six groups (n = 8): control, model, silymarin, TFC low dose (TFC-L), TFC medium dose (TFC-M) and TFC high dose (TFC-H) groups. Mice in the control group were fed a Lieber-DeCarli control diet consisting of 35% fat, 18% protein and 47% carbohydrate, while mice in the other groups were fed a liquid diet containing 5% ethanol consisting of 35% fat, 18% protein, 11% carbohydrate and 36% alcohol. Each mouse was fed 30 mL per day (including any wastage during feeding) and no water was given during this period. The ethanol concentration was gradually increased from 1% to 5% over 5 days and then 5% ethanol was used for the next 22 days. During the next 3 weeks, the TFC low, medium and high dose administration groups were given daily gavage of TFC extract at concentrations of 50 mg/kg, 100 mg/kg and 200 mg/kg respectively, while the positive group was given 36.4 mg/kg of silymarin solution by gavage per day. To simulate the drinking habits of people with drinking problems, mice in the model and dosing groups were gavaged with 31.5% (*v/v*) ethanol on the morning of days 11 and 22, and the control group was given isocaloric dextrin maltose by gavage. Fecal samples were collected during the last 3 days, frozen in liquid nitrogen and then stored at −80°C. The final administration of alcohol to the mice was fasted for 9 h and blood was obtained by removing the eyeball. Liver and colon were removed, immediately frozen in liquid nitrogen and then stored at −80°C until use. The contents of the cecum were collected in sterile tubes and stored as described above.

### 2.4 Biochemical parameters

Serum is obtained by centrifugation of blood at 4°C at 3,000 r/min. Liver homogenates were obtained by homogenising liver tissue in pre-cooled saline at a mass to volume ratio of 1:9, followed by centrifugation at 3,000 r/min for 15 min at 4°C.

#### 2.4.1 Analysis of serum biochemical parameters

ALT and AST in mice serum were measured by the Wright’s method. TC and TG in mice serum were measured by the COD-PAP and GPO-PAP methods respectively, using the appropriate test kits according to the instructions. During the operation, serum was diluted according to the results of the pre-test or accordingly.

#### 2.4.2 Determination of oxidative stress related indexes in liver

SOD, GSH, CAT, and MDA levels in the liver homogenate supernatant were determined using commercial colorimetric kits. Total protein values were determined by the BCA protein quantification kit. During manipulation, homogenates were processed according to the results of pre-experiments or diluted accordingly.

#### 2.4.3 Measurement of inflammation-related indicators

The values of LPS in serum and TNF-α, IL-1β and IL-6 in liver or colon tissues were measured by ELISA kits. Total protein levels were measured by the BCA protein quantification kit.

### 2.5 Histological examinations

Liver or colon tissues were immersed in 4% paraformaldehyde solution for 24 h. The samples were dehydrated, embedded in paraffin, cut into thin slices (4 μm) and the paraffin sections were stained with hematoxylin-eosin staining. Finally, the pathological changes of liver and colon tissues were observed by light microscopy.

Fresh liver tissue was immobilized in 4% paraformaldehyde solution for 24 h and then embedded with OTC embedding agent. After rapid freezing, the tissue sections were cut (8–10 μm) and stained with oil red O. The degree of hepatic steatosis was observed under an optical microscope at ×200 magnification.

### 2.6 Western blot

The total protein was extracted from liver and colon tissues using RIPA buffer (Servicebio) and concentrations were detected using a BCA protein analysis kit (Servicebio). Proteins from the different groups of samples were separated by electrophoresis using SDS-PAGE gels transferred onto PVDF microporous membranes. The transferred membranes were placed in an incubation bath with TBST and washed once quickly, then skimmed milk was added and placed on a decolorization shaker and closed for 30 min at room temperature; primary antibodies were added and incubated at 4°C for 24 h with slow shaking; after washing the membranes three times with TBST, the membranes were incubated with secondary antibodies for 2 h at room temperature and again with TBST for rapid elution; finally the blots were visualized using ECL reagents Visualization. Protein band densities were analyzed by ImageJ software.

### 2.7 Determination of short-chain fatty acids (SCFAs) in cecum contents

In our study, the levels of acetic, propionic, butyric, isobutyric, valeric and isovaleric acids in the cecum were measured. The contents of cecum (0.2 g) were mixed in sterilised water (1.6 mL), then mixed and shaken for 2 min, left to stand at room temperature for 20 min and then centrifuged at 10,000 rpm for 10 min at 4°C. The supernatant was removed. The remaining mixture and sterilised water (1.6 mL) were mixed uniformly, and the above operation was repeated. Next, the supernatant (2 mL) was mixed with the 50% sulphuric acid solution (0.2 mL) and ether (2 mL). Then the mixture was centrifuged at 10,000 rpm for 10 min at 4°C. Finally, the sample consists of the upper layer of ether solution (900 μL) and 2-ethylbutyric acid (100 μL). The concentration of SCFAs was determined by GC-6890 gas chromatography (Agilent Technologies Corporation Ltd, Beijing, China).

### 2.8 Gut microbiota analysis

**FIGURE 1:**
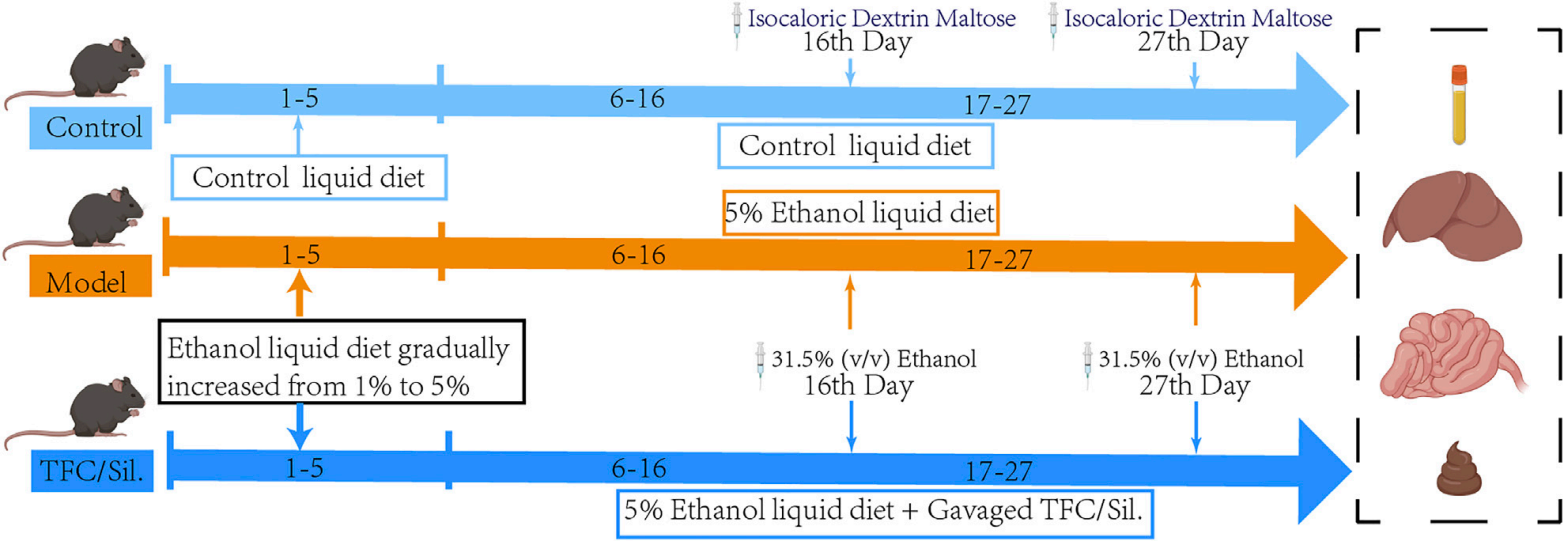
Illustration summarizes the experimental protocol. Liquid feed acclimatisation was carried out on days 1–5, with increasing alcohol content in the alcoholic feed feeding group. At the end of acclimatisation, the model group, each administration group was given a daily liquid feed with 5% alcohol content and gavaged with 31.5% (v/v) ethanol on days 16 and 27, during which time different doses of Sil. And TFC were gavaged daily.

The DNA of fecal samples was extracted by CTAB/SDS method, and the DNA after extracted and purified was selected by PCR for amplification analysis in the V3−V4 highly variable region of 16SrDNA. PCR products were detected by electrophoresis with 2% agarose gel, and the target fragments were recovered and purified. TruSeq^®^ DNA PCR-free Sample Preparation Kit was used for library construction of the purified PCR products. The constructed library was quantified by Qubit and Q-PCR. Double-terminal sequencing of qualified libraries was performed on Illumina NovaSeq sequencing platform (Novogene Bioinformatics Technology Co., Ltd, Beijing, China). Finally, the sequences were clustered into OTUs (Operational Taxonomic Units) with 97% consistency, and the OTUs sequences were annotated with different levels of species for subsequent analysis (Figure 1).

### 2.9 Statistical analysis

All data were expressed as mean ± SD. Statistical analysis was performed using SPSS v20.0 software. Differences in parametric data between the control and model groups were analysed by *t*-test; differences in parametric data between the model and administration groups were determined by one-way analysis of variance (ANOVA). Changes were considered significant if *p* < 0.05 (two-tailed).

## 3 Results

### 3.1 Effect of TFC on body weight and liver index of mice with alcoholic liver injury

**FIGURE 2:**
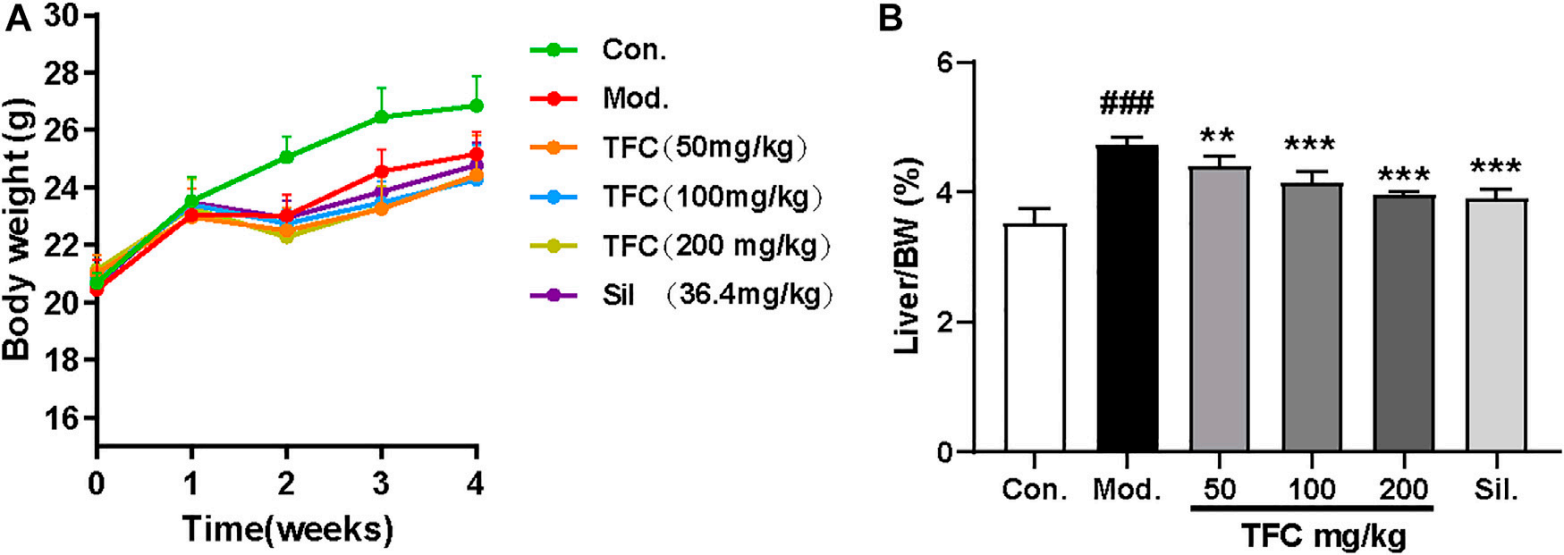
**(A)** Body weight **(B)** liver index. Data are expressed as mean ± S.D. ^***#***^ *p* < 0.05, ^***##***^ *p* < 0.01, ^***###***^ *p* < 0.001 vs. control group; ^*^ *p* < 0.05, ^**^ *p* < 0.01, ^***^ *p* < 0.001vs. model group.

The liver index is calculated as the ratio (%) of liver weight (g) to body weight g). During the first stages of ALD, the high calorie consumption associated with excessive alcohol intake leads to a dramatic increase in liver index, as the gain of liver weight is more rapid than the gain of body weight; therefore, the liver index is an informative ratio for diagnosing ALD. Control or alcohol-treated mice were treated with TFC, Sil or vehicle, and their body weights were monitored. At the beginning of the experiment and after 1 week of acclimatization feeding, all mice were found to have similar initial body weights. However, after 1 week, alcohol was added to the diets of some mice. The mice in alcohol diet groups showed a tendency to lose weight during the first week of treatment, which may be due to anorexia symptoms of anorexia caused by the high concentration of alcohol in the liquid feed. However, after 1 week, the alcohol-fed mice began to show a tendency to gain weight, possibly due to the increased calorie intake; treatment with TFC and Sil alleviated this weight gain caused by excessive alcohol consumption. (Figure 2A).

At the end of the entire modeling cycle, the mice were sacrificed, and the liver indexes were determined. The liver index of the alcohol model group was significantly higher than that of the control group; all groups receiving treatment had significantly lower indexes as compared to the model group (Figure 2B). Therefore, both Sil and TFC had a protective effect on the liver; the fact that these liver indexes were lower even than that of the model group suggested that TFC helped to reduce alcohol-induced liver injury.

### 3.2 TFC alleviates liver damage caused by alcohol intake

**FIGURE 3:**
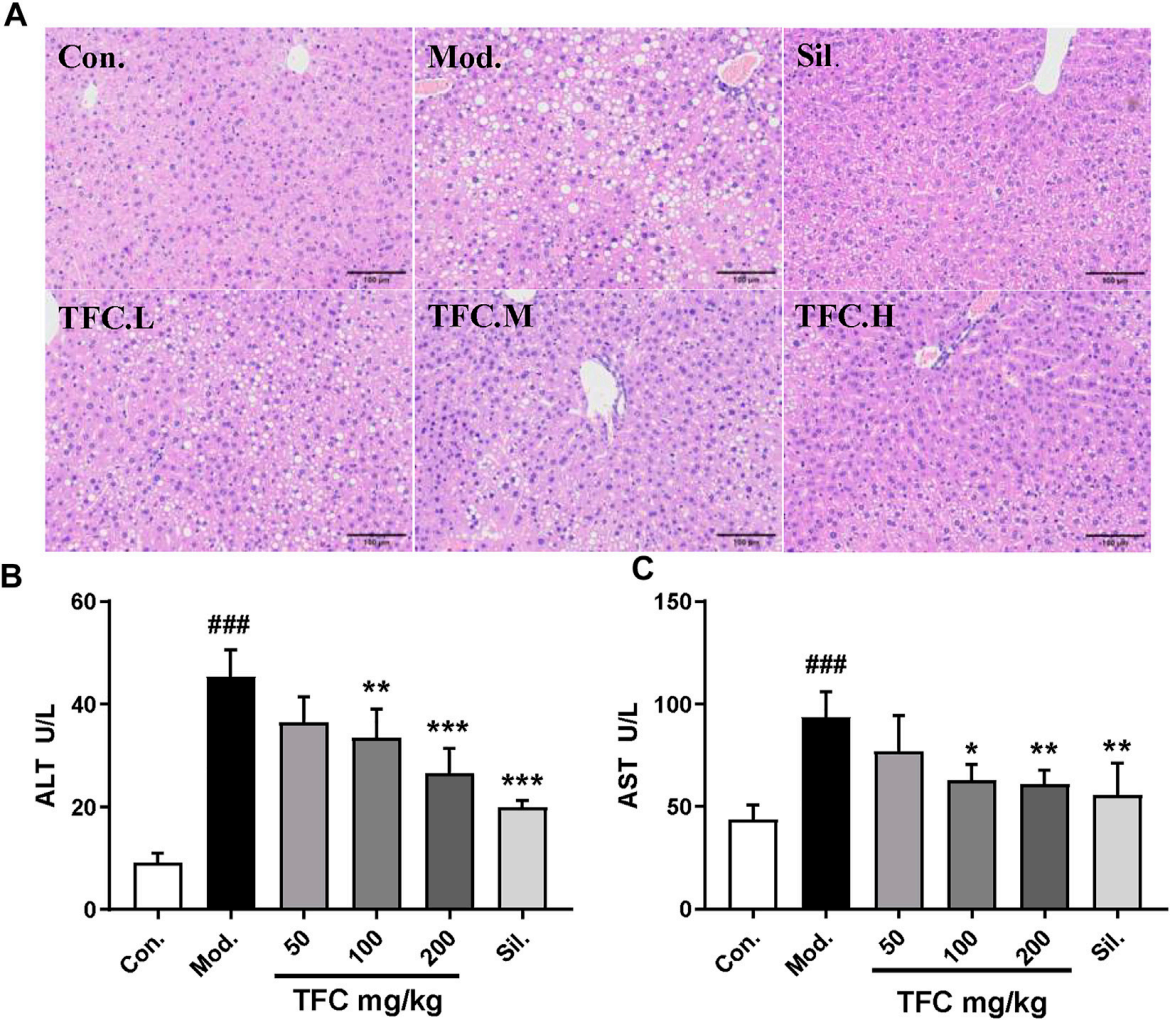
TFC alleviates liver damage caused by alcohol intake **(A)** Histological changes of liver sections measured by HE staining at 200× magnificati **(B)** serum ALT **(C)** serum AST. Data are expressed as mean ± S.D. ^***#***^ *p* < 0.05, ^***##***^ *p* < 0.01, ^***###***^ *p* < 0.001 vs. control group; ^*^ *p* < 0.05, ^**^ *p* < 0.01, ^***^ *p* < 0.001vs. model group.

In order to evaluate the effects of TFC on liver injury induced by alcohol, the morphological changes of liver and the biochemical indices of serum were investigated. HE staining (Figure 3A) showed that the morphology and structure of the hepatocytes in the control group were intact and that there was no detectable steatosis. In the model group, significant changes in liver tissue structure were observed, the cell arrangement was disordered, the cell spacing was increased, and many lipid vesicles of different sizes appeared in the cytoplasm. These changes are consistent with strong hepatocyte steatosis, accompanied by inflammatory cell infiltration. Histopathological changes in the livers of mice given 50, 100 and 200 mg/kg of TFC by gavage showed varying degrees of improvement relative to the livers of control mice. Histopathological examination showed that after administration of all doses of TFC, especially high doses of TFC, significantly ameliorated liver damage in mice with ALD.

To evaluate the degree of liver damage, the serum ALT and AST levels were determined. As shown in Figures 3B, C, as compared with the control group, the activities of AST and ALT in the model group were significantly increased (*p* < 0.01), suggesting the occurrence of liver injury. Treatment with TFC mitigated these elevations in a dose-dependent manner, especially at high doses (200 mg/kg).

### 3.3 TFC regulates indicators of oxidative stress in liver tissue

**FIGURE 4:**
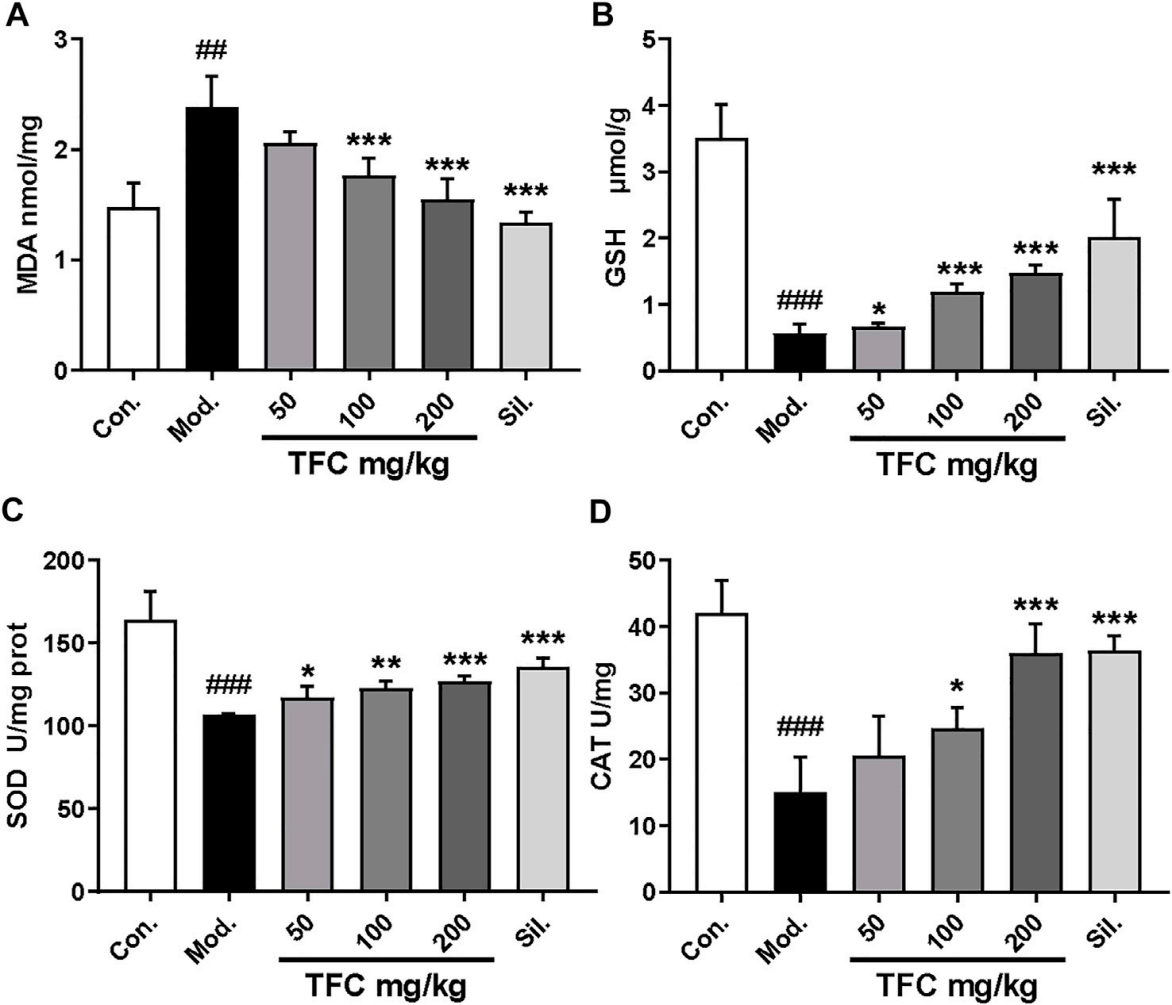
TFC enhanced antioxidant capacities in ALD rat. Hepatic levels of MDA **(A)**; GSH **(B)**; SOD **(C)**; CAT **(D)**. Data are expressed as mean ± S.D. ^*#*^ *p* < 0.05, ^*##*^ *p* < 0.01, ^*###*^ *p* < 0.001 vs. control group; ^*^ *p* < 0.05, ^**^ *p* < 0.01, ^***^ *p* < 0.001vs. model group.

Since oxidative damage is another factor that leads to ALD, the effect of TFC on parameters indicative of the hepatic oxidative stress response (MDA, GSH, SOD and CAT) was determined (Figure 4A-D) (Figure 4). In the model group fed with an alcohol-containing liquid diet, CAT, SOD and GSH indexes were markedly reduced (*p* < 0.01), while the level of the lipid peroxide MDA was significantly increased (*p* < 0.01). On the contrary, these liver oxidative stress parameters were significantly improved upon administration of TFC, especially in the high-dose group, suggesting that TFC can protect liver cells against oxidative stress induced by alcohol consumption.

### 3.4 TFC suppresses lipogenesis

**FIGURE 5:**
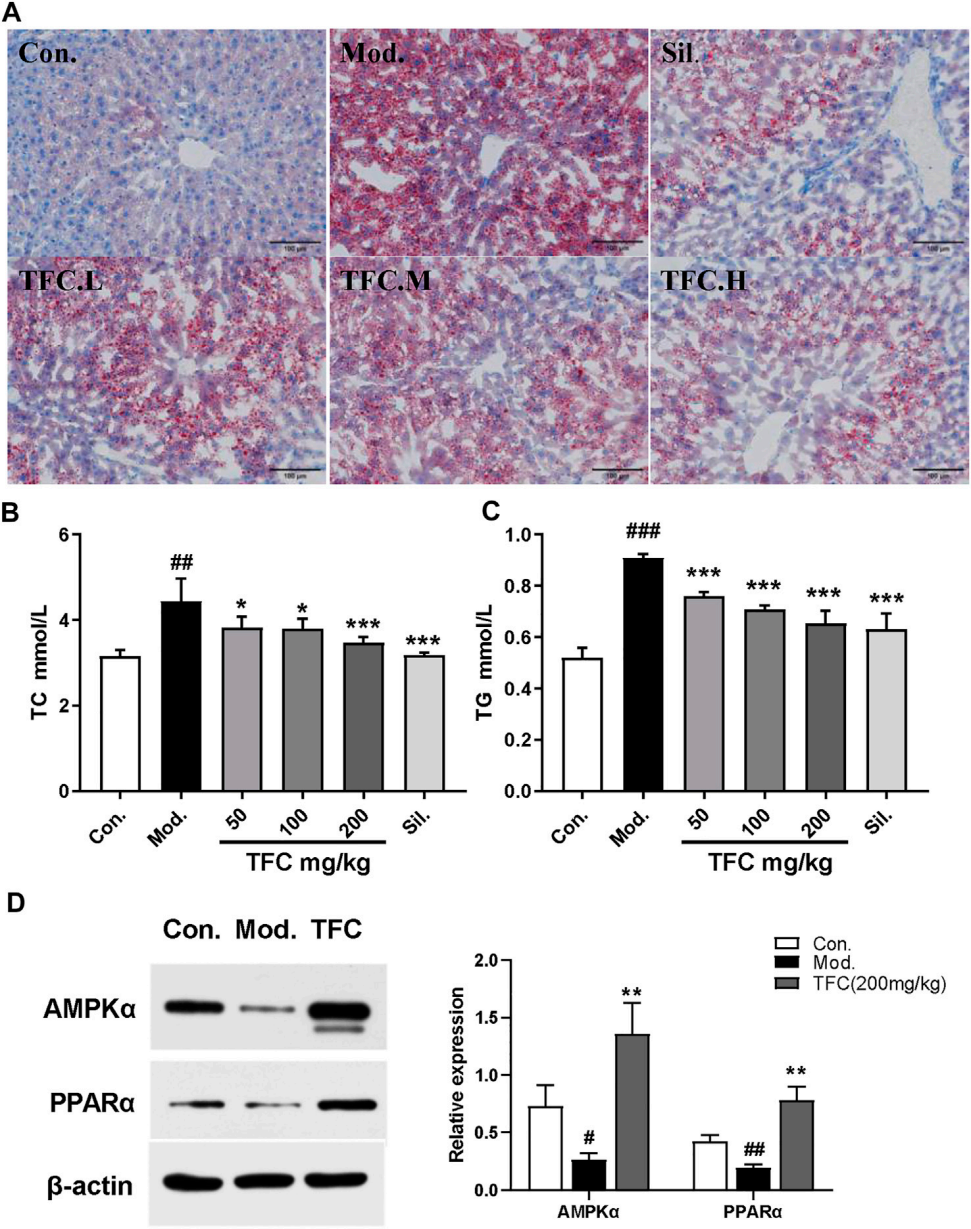
TFC suppressed lipogenesis **(A)** Oil Red O staining showing the accumulation of fat in the liver (200magnififications) **(B)** serum level of TC **(C)**serum level of TG **(D)** protein expression of AMPKα and PPARα in the liver. Data are expressed as mean ± S.D. ^*#*^ *p* < 0.05, ^*##*^ *p* < 0.01, ^*###*^ *p* < 0.001 vs. control group; ^*^ *p* < 0.05, ^**^ *p* < 0.01, ^***^ *p* < 0.001vs. model group.

Hepatic steatosis is the dominant feature in the pathogenesis of ethanol-induced liver injury (Gao and Bataller, 2011). To evaluate the effects of TFC on hepatic steatosis induced by ethanol exposure, hepatic lipid accumulation was qualitatively and quantitatively examined by Oil Red O staining and quantification kits, respectively. The results of Oil Red O staining (Figure 5A) showed that the hepatocyte nucleus in the control group was blue, no obvious red lipid droplets were found in the hepatocytes, and the hepatocyte gap was clear. In contrast, in the model group, hepatocytes were obviously enlarged, and diffuse red granular lipid droplets appeared in the visual field. After administration of TFC for three consecutive weeks, all dose groups, especially the TFC-high dose group, exhibited improved indexes of lipid accumulation of hepatocytes in mice with ALD. According to the Oil Red O staining results, we further explored changes to lipid metabolism-related parameter indexes (TC and TG) in serum. As demonstrated in Figures 5B, C, the model group had higher serum levels of TC and TG, and these increases were significantly ameliorated (*p* < 0.05) upon treatment with TFC.

To further reveal the mechanisms of the suppression of lipogenesis by TFC, the expression of lipogenesis-related proteins in mice was determined. The proteins that were investigated were AMPKα, which plays a crucial role in regulating hepatic lipid metabolism, and PPARα, which modulates the expression of genes involved in hepatic lipid metabolism (Preidis et al., 2017). We thus investigated whether TFC restores alcohol diet-induced imbalances to lipid metabolism by regulating pathways involving AMPKα or PPARα. As presented in Figure 5D, the model group exhibited significantly decreased levels of AMPKα and PPARα (*p* < 0.05) relative to the control group. In the ALD groups receiving TFC treatment, a significantly higher expression of AMPKα and PPARα (*p* < 0.01) was observed relative to the model group. This demonstrates that TFC regulates the expression of AMPKα and PPARα proteins and inhibits lipid accumulation in the liver.

### 3.5 TFC ameliorates alcohol-induced hepatic-intestinal inflammatory responses *via* the TLR4/NF-κB pathway

**FIGURE 6:**
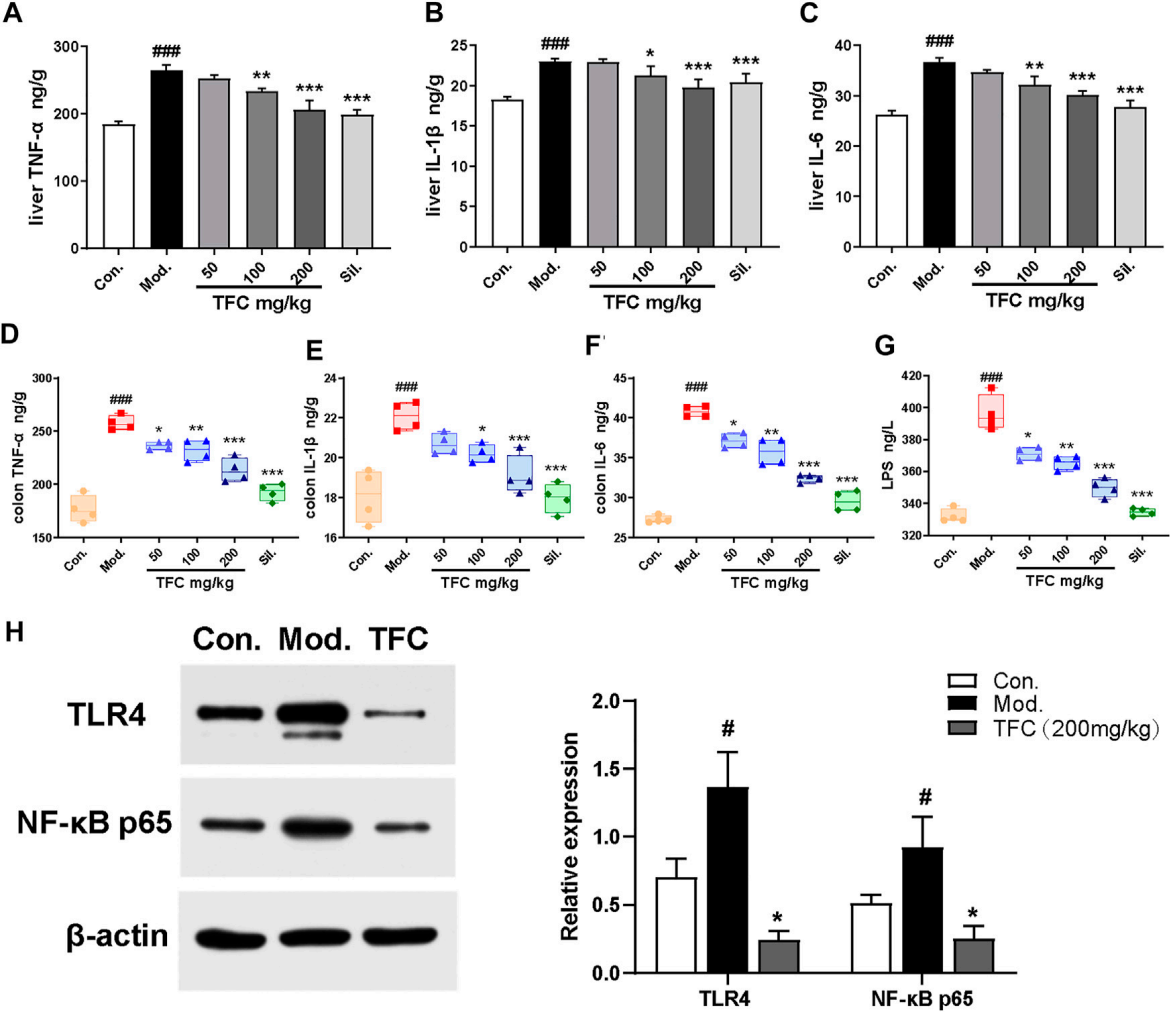
TFC ameliorates alcohol -induced hepatic-intestinal inflammatory response via the TLR4/NF-κB pathway **(A)** Level of TNF-α in liver **(B)** level of IL-1β in liver **(C)** level of IL-6 in liver **(D)** level of TNF-α in colon **(E)** level of IL-1β in colon **(F)** level of IL-6 in colon **(G)** level of LPS in serum **(H)** the expression levels of TLR4 and NF-κB p65 were detected by western blot analysis. Data are expressed as mean ± S.D. ^*#*^ *p* < 0.05, ^*##*^ *p* < 0.01, ^*###*^ *p* < 0.001 vs. control group; ^*^ *p* < 0.05, ^**^ *p* < 0.001, ^***^ *p* < 0.001vs. model group.

Numerous studies have shown that chronic alcohol consumption can trigger an inflammatory response; therefore, the effects of alcohol and TFC on pro-inflammatory factor in mice were evaluated. As revealed in Figure 6, the levels of TNF-α, IL-1β, and IL-6 in the liver (Figure 6A-C) and intestinal tissues (Figure 6D-F) of the model mice were significantly increased relative to control, while TFC at medium and high doses significantly reduced this increase.

A growing body of evidence has implicated Kupffer cells activated by gut-derived LPS *via* TLR4-mediated NF-κB signaling in the pathogenesis of alcohol-induced hepatic inflammation (Ding et al., 2012). As shown in Figure 6G, our analyses demonstrated that the serum levels of LPS were significantly higher in the model group compared with the control group (*p* < 0.01). After TFC treatment, especially at higher TFC doses, the increase of LPS was significantly inhibited.

Meanwhile, the levels of TLR4/NF-κB signaling pathway-related proteins in the livers of each group were measured. Compared with the control group, TLR4 and NF-κB protein expression levels were significantly increased in the model group, while TFC intervention suppressed the increase in the expression of these proteins (Figure 6H). Taken together, our results demonstrated that TFC attenuated the inflammatory response induced by alcohol consumption by inhibiting the TLR4/NF-κB signaling pathway, and it exerted a protective effect against liver injury caused by alcohol intake.

### 3.6 TFC attenuates intestinal damage caused by alcohol intake

**FIGURE 7:**
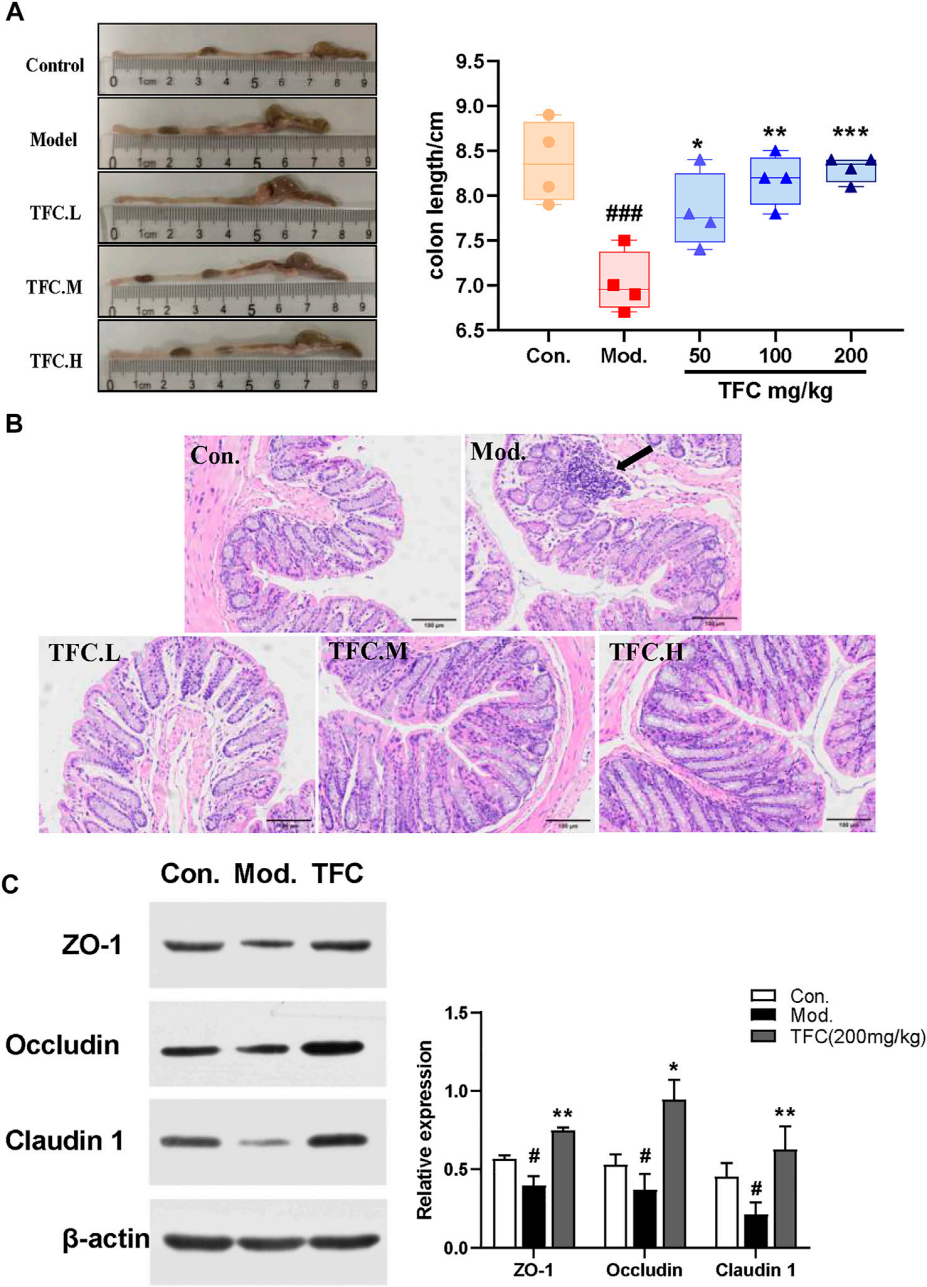
TFC attenuates intestinal damage caused by alcohol intake **(A)** Representative images of colon morphology and length **(B)** histological changes of liver sections measured by HE staining at ×200 magnification **(C)** the representative blots of relative ZO-1, Occludin and Claudin one in the colon with β-actin as a loading control; Data are expressed as mean ± S.D. ^*#*^ *p* < 0.05,^*##*^ *p* < 0.01,^*###*^ *p* < 0.001 vs. control group;^*^ *p* < 0.05, ^**^ *p* < 0.01, ^***^ *p* < 0.001vs. model group.

As shown in Figure 7A, it was found that the colon lengths of mice in the model group were significantly shorter than those in the control group, and that TFC intervention improved the occurrence of this condition. To further assess the effect of TFC on the colons of mice, HE staining of colon tissue was performed. Histological analysis showed that, as compared with the control group, ethanol-fed mice exhibited cell lysis deformation, a loose arrangement of intestinal glands, local lymphocyte aggregation in the mucosal layer sparse and atrophic surrounding villi, and structural destruction of villi (Figure 7B). In contrast, TFC treatment reduced these morphological and pathological changes, especially at the highest dose of 200 mg/kg.

To determine whether TFC affects the intestinal barrier in alcohol-treated mice, we measured the expression of claudin-1, occludin, and ZO-1 proteins. As shown in Figure 7C, the reduced expression levels of claudin-1, occludin, and ZO-1 proteins in the model group indicated that the intestinal barrier of mice was damaged by alcohol intake, whereas the pre-treatment with TFC reversed this phenomenon, demonstrating that TFC improves the intestinal damage caused by alcohol consumption.

### 3.7 TFC regulates the concentration of short-chain fatty acids in the cecum

**FIGURE 8:**
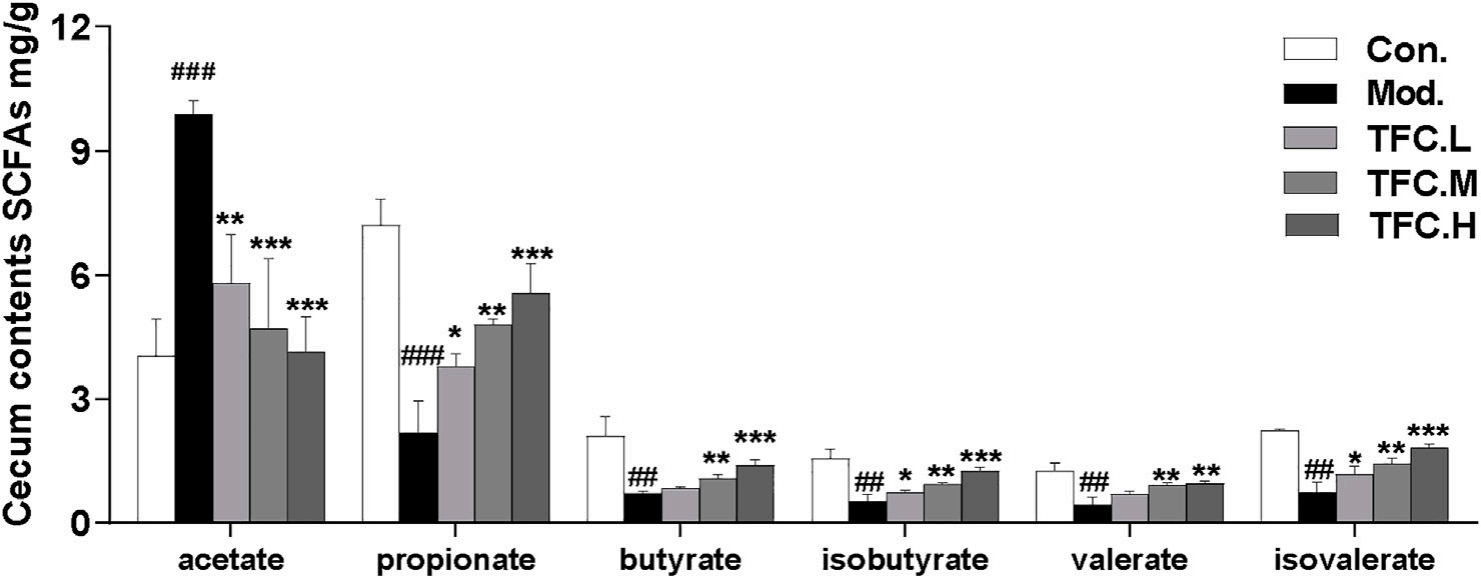
Content of SCFAs in cecum contents. The data are expressed as the means ± SD. Data are expressed as mean ± S.D. ^*#*^ *p* < 0.05, ^*##*^ *p* < 0.01, ^*###*^ *p* < 0.001 vs. control group; ^*^ *p* < 0.05, ^**^ *p* < 0.01, ^***^ *p* < 0.001vs. model group.

SCFAs are saturated fatty acids with main chains containing at most six carbons (Cait et al., 2018), and they have been well established as representative metabolites of intestinal flora. In our study, the contents of six short-chain fatty acids in the cecum were measured. As illustrated in Figure 8, the results showed that the acetate content in cecal contents of rats in the model group was significantly increased, and the acetate content was significantly decreased after TFC administration. The content of all other SCFAs, except for acetate, decreased significantly after excessive alcohol consumption, and these decreases were reversed by TFC administration. In particular, the levels of the remaining five SCFAs increased significantly in the TFC high-dose group relative to the model group.

### 3.8 TFC regulates the structure and composition of gut microbiota

**FIGURE 9:**
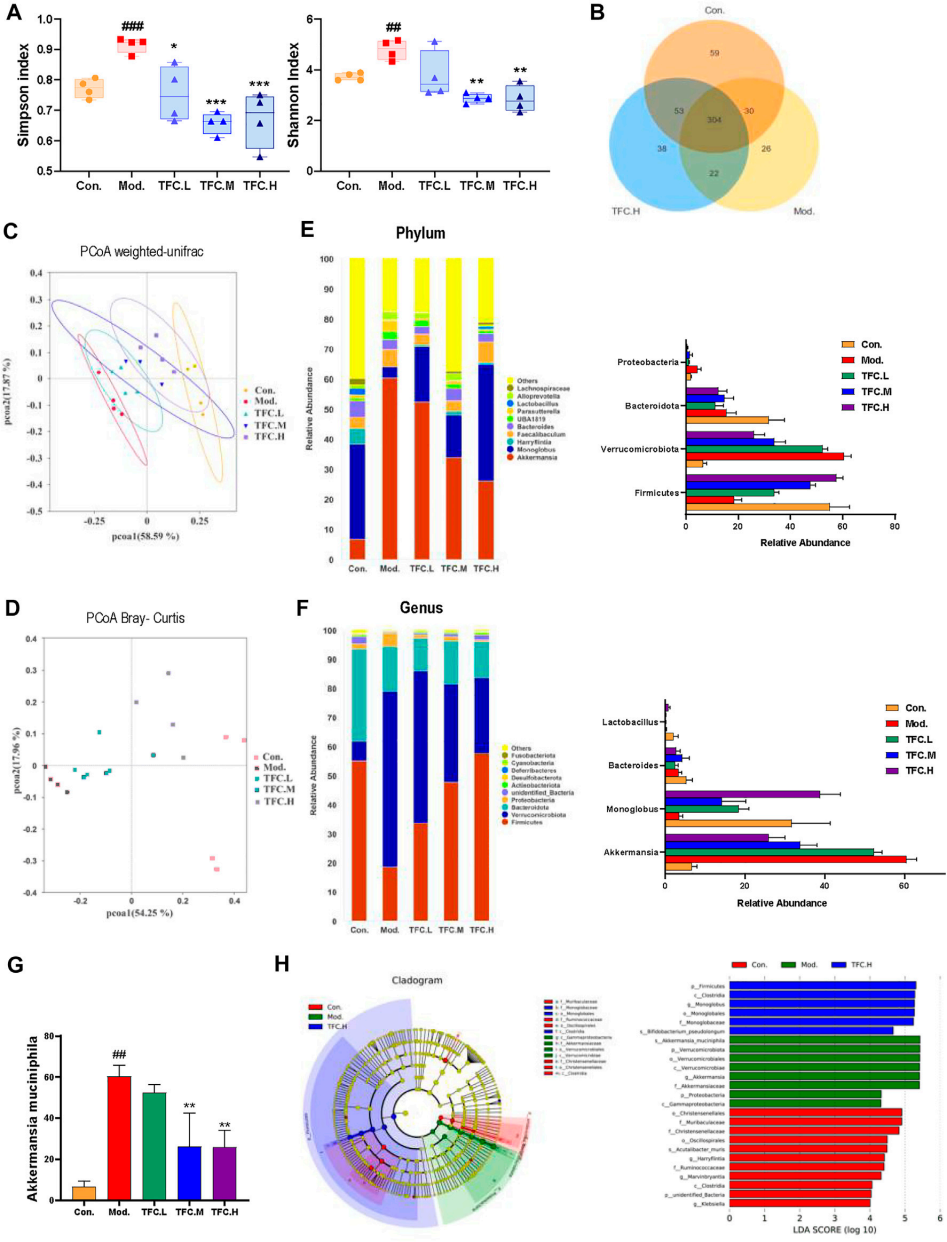
TFC regulates the structure and composition of the intestinal flora **(A)** Shannon and Simpson indices of the gut microbial communities in different groups **(B)** Venn diagrams illustrating the discrepancy of OTUs among the different groups **(C)** PCoA weighted-unifrac **(D)** PCoA Bray-Curtis **(E)** Composition of gut microbiota at the phylum level and the abundance of *Firmicutes*, *Verrucomicrobiota*, *Bacteroidota* and *Proteobacteria* **(F)** Composition of gut microbiota at the genus level and the abundance of *Akkermansia*, *Monoglobus*, *Bacteroides* and *Lactobacillus* **(G)** the abundance of *Akkermansia muciniphila* **(H)** linear discriminant analysis (LDA) effect size (LEfSe) analysis was used to identify the most differentially abundant taxa of bacterial communities. Data are expressed as mean ± S.D. ^*#*^ *p* < 0.05, ^*##*^ *p* < 0.01, ^*###*^ *p* < 0.001 vs. control group; ^*^ *p* < 0.05, ^**^ *p* < 0.01, ^***^ *p* < 0.001vs. model group.

**FIGURE 10:**
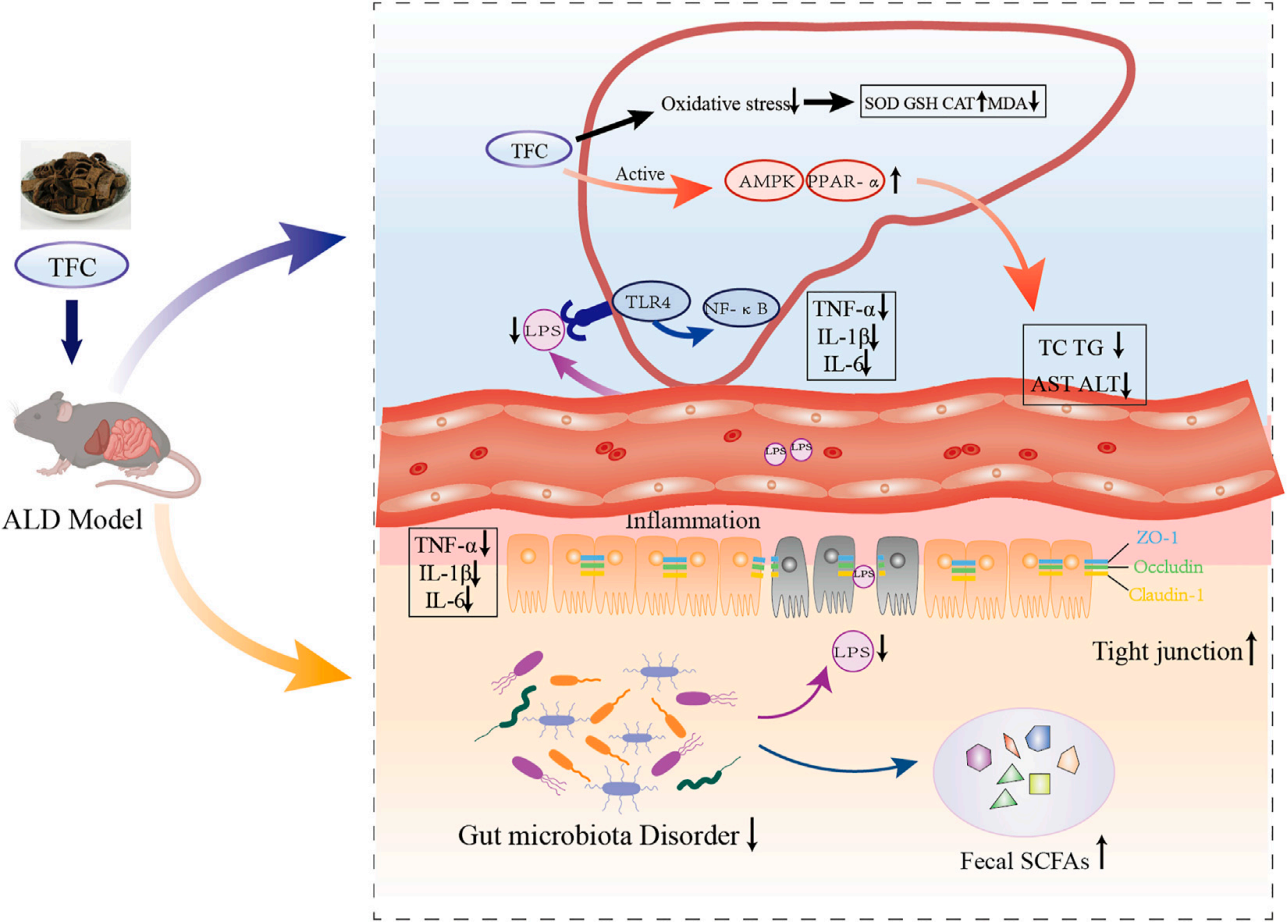
Schematic illustration on mechanism of action of TFC against alcoholic liver disease based on “gut-liver axis”. TFC reduces oxidative stress caused by ALD, activates the AMPK/PPARa pathway to reduce lipid accumulation, regulates intestinal flora disorders caused by ALD, restores SCFAs in the cecum, and reduces inflammatory responses in the liver and intestine *via* “gut-liver axis”.

To explore the possible mechanisms by which TFC exerts its medicinal effects, a study comparing the gut microbiomes of control and model mice to those treated with high-dose TFC was conducted. As shown in Figure 9A, the Shannon and Simpson indexes were significantly increased in the model group compared to the control group, implying that ingestion of alcohol led to an increase in the diversity of the gut microbiota, while TFC treatment reversed this trend. Furthermore, a Venn diagram analyses showed a total of 304 OUTs in the control, model, and TFC high-dose groups, indicating the presence of a strong core microbiota. In addition to the 304 common OUTs, 53 OUTs were shared by the control group and the TFC high-dose group, while only 22 OUTs were shared by the TFC high-dose group and the model group. The control group had the highest number of unique OUTs (59), followed by the TFC high dose group (38) and the model group (26) (Figure 10) (Figure 9B).

A Bray Curtis-based Principal Coordinate Analysis (PCoA) plot clearly shows that the distance between the TFC high-dose and control groups is much smaller than the distance between the TFC high-dose and model groups (Figure 9C). In addition, the PCoA plot based on a weighted UniFrac analysis shows that the model and control groups are located on either side of each other, indicating that there is a significant difference in the composition of the flora between the two groups (Figure 9D). Compared to the model group, the intestinal microbes of the mice in the TFC high dose group are more closely related to those in the control group. The above data suggest that TFC alters the changes to the intestinal microbiota that are caused by alcohol intake.

This study also compared specific gut microbes between different groups of mice at the family, genus and phylum levels to provide insight into the regulatory effects of TFC on gut microbiota. At the phylum level (Figure 9E), the relative abundance of Firmicutes decreased and the relative abundance of Verrucomicrobiota increased in the model group compared to the control group, and TFC reversed the trend in a dose-dependent manner. Although the abundance of *Bacteroidota* decreased in the model group, TFC did not have an effect on bacteria of this phylum.

At the genus level (Figure 9F), compared with the control group, the relative abundance of *Akkermansia* was increased, and the relative abundance of *Monoglobus* was decreased in the model group, but the relative abundances of bacteria of these genera were reverted to the level of control by treatment with high dose TFC. The decrease in the relative abundance of *Bacteroides* in the model group was reversed by gavage administration of TFC. In addition, species of *Lactobacillus* in the model group almost disappeared, but a small number *of Lactobacillus* species were observed in the TFC high dose group.

Finally, at the species level (Figure 9G), TFC reduced the levels of *Akkermansia muciniphila* in a dose-dependent manner, bringing the levels of this species closer to the control than to the model mice. From the results of a Lefse analysis, it can also be seen that the most abundant differential phylum in the TFC group is Firmicutes, mainly including Monoglobaceae and Monoglobales, while Proteobacteria, Verrucomicrobiota were more abundant in the model group, mainly including Akkermansiaceae, Verrucomicrobiae and Verrucomicrobiales (Figure 9H).

## 4 Discussion

ALD is the second most common cause of death in humans. Oxidative stress, hepatic steatosis, inflammatory response and disturbance of the intestinal flora are all key factors in the pathogenesis of ALD. At present, there is no universally accepted treatment for ALD, so it is necessary to explore new therapeutic drugs. The major components of the TCM TFC include quercetin, kaempferol, naringenin, and glycosides, which are flavonoids that have been shown to exert anti-inflammatory, anti-tumor, hypoglycemic, lipid-lowering, anti-coagulation, and liver protective effects (Kim et al., 2018; Tang et al., 2019; Wang et al., 2020; Alshehri et al., 2021, 1). The aim of this study was to investigate the possible mechanisms of TFC in the treatment of ALD through analyses of lipid metabolism and changes in gut microbes *in vivo*.

The liver is an important organ for alcohol metabolism. Chronic exposure to alcohol or exposure to excessive amounts of alcohol first results in changes of serum ALT and AST, which reflect liver function. In addition, the progression to ALD is characterized by an increased liver index. Our results showed that TFC significantly reduced the serum ALT and AST levels that were elevated after alcohol consumption. Histological analysis of liver sections stained with HE further confirmed the hepatoprotective effect of TFC, which alleviated steatosis and vacuolar degeneration of liver tissue in a dose-dependent manner. The above data indicated that the mouse model of ALD was successfully established and that TFC has a protective effect on ALD.

Oxidative stress plays an important role in the pathogenesis of ALD. Long-term heavy drinking leads to a surge of reactive oxygen species in the liver, which consumes a large number of antioxidants, including GSH and SOD, resulting in an imbalance in the oxidative balance of the body and causing oxidative stress (Yu et al., 2016). Excess free radicals react with lipids in a lipid peroxidation reaction, producing MDA. Excessive accumulation of MDA can damage the structure and function of cell membranes, leading to cell necrosis and apoptosis (Sh et al., 2018). SOD is a natural endogenous antioxidant enzyme that scavenges superoxide radicals and can promote the production of H_2_O_2_ and O_2_, while CAT hydrolyzes the H_2_O_2_ produced by SOD, thus reducing the number of superoxide anion radicals in the body and inhibiting the lipid peroxidation reaction. Hence, the levels of SOD, CAT, MDA, and GSH in the liver are several important indicators for the evaluation of the liver alcoholic oxidative damage. In this study, TFC was shown to alleviate the elevated MDA levels caused by alcoholic liver injury in mice, and to increase SOD, CAT and GSH activities to accelerate lipid metabolism and oxidative breakdown *in vivo*. Thus, TFC was shown to protect the liver by reducing oxidative stress and improving antioxidant capacity.

Hepatic steatosis is the most common symptom following an early diagnosis of ALD. AMPK, as a major energy metabolism sensor, is involved in maintaining intracellular energy metabolism homeostasis, and it plays an important role in regulating fatty acid synthesis and metabolism (Srivastava et al., 2012). AMPK also upregulates PPARα expression to promote fatty acid oxidation, thereby inhibiting lipid accumulation. PPARα is mainly expressed in the liver, and its expression improves hepatic lipid accumulation, inflammatory responses and fibrosis. Excess alcohol in the body inhibits fatty acid oxidation by suppressing the transcriptional activity and DNA binding capacity of PPARα (A et al., 2001). This study demonstrated that long-term feeding of an alcoholic liquid diet to mice reduced the expression of AMPK and PPARα, accelerated alcoholic steatosis, and increased serum levels of TC and TG. In addition, Oil Red O-stained liver sections revealed lipid accumulation in the liver tissue of these model mice. Treatment with TFC, on the other hand, activated AMPK and PPARα expression, promoted lipid metabolism and alleviated the hepatic lipid accumulation caused by alcohol.

The integrity of the intestinal barrier protects the body from harmful components of the gut, such as microorganisms and their metabolites. An important role is played by the tight junctions located at the apical part of the intestinal epithelium, which are composed of transmembrane proteins, including claudins and occludins, peripheral membrane proteins, including ZO-1 and ZO-2, and regulatory proteins. Several studies have demonstrated that alcohol and its degradation products disrupt intestinal tight junctions (Cassard and Ciocan, 2018). In the present research, the expression of ZO-1, occludin and claudin-1 proteins was found to be significantly reduced in the model group fed only the Lieber-DeCarli alcohol liquid diet, while TFC administration led to a significant upregulation of the expression of tight junction proteins.

The disruption of the intestinal flora caused by heavy alcohol consumption can lead to the production of harmful bacteria or harmful substances such as endotoxins. Due to the damaged intestinal barrier and increased intestinal permeability, LPS can translocate to the plasma and liver. LPS entering the liver through the portal blood stream binds to endotoxin receptors and TLR-4 and activates Kupffer cells. The activated receptors promote the transcription of various genes encoding various cytokines and chemokines in these cells by activating NF-κB or other transcription factors, thereby releasing large amounts of oxygen radical-generating cytokines and inflammatory mediators such as TNF-α, IL-1β, and IL-6. Together, these effects lead to a potent inflammatory response. In the present study, both TLR4 and NF-κB expression levels were higher in the livers of model mice, and further measurements of TNF-α, IL-1β and IL-6 levels in liver and intestinal tissues also showed an increasing trend. The TLR4/NF-κB protein expression levels and elevated inflammatory factor levels were reduced by TFC supplementation. This suggests that TFC may attenuate the inflammatory response caused by excessive alcohol consumption by protecting the integrity of the intestinal barrier and inhibiting the activation of the TLR4/NF-κB signaling pathway by intestine-derived LPS.

Alcohol abuse can lead to overgrowth and imbalance of bacteria in the small and large intestine. Overgrowth of intestinal bacteria is defined as an increase in the number of intestinal bacteria, especially harmful bacteria, which can further lead to dysbiosis, which is a key reason for the development of ALD. Therefore, we examined the gut microbiota composition of ALD mice by 16S rRNA sequencing analysis. In our study, mice in the alcohol-fed model group had elevated a-diversity and a significant increase in Simpson’s and Shannon’s indices as compared to the control group, which is consistent with other studies (H et al., 2018, 2021). The results of the β-diversity analysis showed that the TFC intervention regulated the intestinal ecosystem to levels similar to those of control group. Bull-Otterson et al. reported that heavy alcohol consumption led to a decrease in *Bacteroidetes* and *Firmicutes* as well as an increase in Proteobacteria, in keeping with our findings (Bull-Otterson et al., 2013). *Proteobacteria* have been shown to be significantly negatively correlated with tight junction proteins and significantly positively correlated with markers of liver inflammation and oxidative stress (Xia et al., 2020). *Lactobacillus* is the main beneficial bacteria in the intestinal tract. *Lactobacillus* supplementation has been shown to not only reduce endotoxemia, but also to improve intestinal leakage and liver inflammation in ALD patients (Meroni et al., 2019). In our study, *Lactobacillus* basically disappeared from the feces of mice in the model group, and its levels were found to increase upon gavage administration of high-dose TFC. *Akkermansia muciniphila* plays an important role in metabolic diseases, and several studies have demonstrated that *A. muciniphila* may be associated with obesity and intestinal immunity. Kim et al. found that treatment with *A. muciniphila* prevented fatty liver disease in obese mice (Kim et al., 2020). Other studies on ALD have reported a decrease in *A. muciniphila* in the intestines of mice administered alcohol by gavage (Jiang et al., 2021). Not all studies have reported a decrease in *A. muciniphila* after alcohol administration, and changes in populations of this bacterium may be influenced by the amount of alcohol ingested. A study by found increased levels of *A. muciniphila* in the intestine of mice fed a 2.6% Lieber-DeCarli diet (Caslin et al., 2019). Similarly, found that short-term alcohol consumption (0.8 g/kg gavage for 5 days) increased *A. muciniphila* levels in mice (Lee et al., 2020). These studies suggest that acute alcoholic liver injury may lead to increased levels of *A. muciniphila*. SCFAs are products of bacterial fermentation, and dysbiosis of the intestinal flora may lead to differences in the products of intestinal fermentation. These fatty acids are essential for maintaining the integrity of the intestinal barrier and mucosal immune tolerance and as an energy source for the intestinal epithelium. It was found that following ethanol administration, intestinal levels of most SCFAs were reduced, with the exception of increased levels of acetic acid, which is a metabolite of ethanol (Xie et al., 2013). The decrease in butyric acid, in particular, may further contribute to the development of hepatic steatosis, inflammation and liver injury (Donde et al., 2020). More importantly, butyric acid also maintains the integrity of tight junction proteins, the intestinal barrier, and the stability of the intestinal lining (Baxter et al., 2019). Apart from butyric acid, propionic acid plays an important role in the inhibition of hepatic lipid accumulation mainly through the activation of AMPKα expression (Yoshida et al., 2019). Our study showed that TFC significantly improved the changes in SCFAs that were caused by dysbiosis of the intestinal flora after alcohol consumption, thereby promoting intestinal tight junction expression and regulating hepatic lipid metabolism.

## 5 Conclusion

In general, gavage administration of TFC showed beneficial effects in C57BL/6 mice suffering from ALD. TFC significantly improves the oxidative stress caused by ALD while effectively reducing MDA levels as well as increasing SOD, GSH and CAT levels in the liver. In addition, activation of the AMPK/PPARα pathway by TFC remarkably improved hepatic fat accumulation and reduced serum levels of TC and TG. TFC improved intestinal epithelial permeability and reduced serum LPS levels, which in turn inhibited the TLR4-mediated NF-κB pathway that can lead to liver inflammation and attenuated the inflammatory response caused by alcohol consumption. TFC restored the composition of the intestinal flora, with an increase in the relative abundance of *Lactobacillus*, Bacteroidetes and Firmicutes, and an increase in the relative abundance of Proteobacteria and *Akkermansia muciniphila* decreased. At the same time, TFC enriched the content of SCFAs in the contents of the cecum. These results suggest that TFC prevents alcohol-induced chronic liver injury by modulating the gut-liver axis and the AMPK/PPARα pathway.

## Data Availability

The authors acknowledge that the data presented in this study must be deposited and made publicly available in an acceptable repository, prior to publication. Frontiers cannot accept article that does not adhere to our open data policies.
